# Knowledge, Attitudes, and Practices Two Years after the Start of the COVID-19 Pandemic: A Mixed Methods Study

**DOI:** 10.1155/2024/6636649

**Published:** 2024-02-16

**Authors:** Teresa Botigué, Carla Camí, Laia Selva-Pareja, Olga Masot, Anna Espart, Carme Campoy, Judith Roca

**Affiliations:** ^1^Department of Nursing and Physiotherapy, University of Lleida, Lleida 25198, Spain; ^2^Health Care Research Group (GRECS), Biomedical Research Institute of Lleida, Lleida 25198, Spain; ^3^Health Education, Nursing, Sustainability and Innovation Research Group (GREISI), University of Lleida, Lleida, Spain; ^4^Chair of Development of Health and Sustainability Organizations and Territories (DOTSS), University of Lleida, Lleida 25001, Spain; ^5^African Societies Studies Group (GESA), University of Lleida, Lleida, Spain

## Abstract

**Introduction:**

During the COVID-19 pandemic, there was a need to promote the most adequate behaviors. It is essential to know what aspects were implemented and what needs to be re-enforced.

**Objectives:**

(a) To identify the knowledge and behaviors related with preventive measures, lifestyle habits, sources of information, vaccination, and emotions generated and coping strategies and (b) to explore the personal experiences with respect to the knowledge, attitudes, and practices when facing COVID-19.

**Design:**

A convergent mixed method design. *Setting and Participants*. This study was conducted in the Segrià region (Catalonia, Spain) during the months of January and February 2022, with individuals 18 years old or older.

**Methods:**

Based on variables proposed by the WHO and a bibliographic review, an ad hoc electronic survey was utilized for the quantitative part, analyzed through frequency distribution or central tendency and dispersion measurements. For the qualitative part, two focus groups were analyzed through content analysis.

**Results:**

The participants (*n* QUAN = 1,559; *n* QUAL = 19) were aware about and applied the prevention measures, but when delving into it, deficiencies were detected especially when referring to hand-washing; lifestyles remained healthy; the population trusted the information from professionals (78.8%), but excess of information led to saturation; protection (75.3%) and herd immunity (47.2%) were recognized with vaccination; and the psychological impact (69.5%) was buffered with the activation of positive coping measures (99.1%), although it was maintained in more than half of them.

**Conclusions:**

This study showed that prevention measures must be re-enforced, especially hand-washing. Another revealing aspect was psychological impact, which, although coping measures were utilized, was maintained for another two years in most of them. This evidences the need for an intervention centered on this to guarantee the mental health of the population. *Implications for Nursing Management*. The detection of the current needs of the population provides the information necessary for the design of an adapted intervention and for promoting health education programs to address COVID-19 or other future health situations.

## 1. Introduction

In March, 2020, the World Health Organization (WHO) defined COVID-19 as a global pandemic [1]. This pandemic led to an unprecedented situation, unleashing an important crisis in public health. The outbreak of the virus was a worldwide health threat with consequences at different levels [2]. Faced with this situation, a need arose to rapidly and constantly interact with society to provide information about course of COVID-19 and about the protection or treatment measures at each stage [3]. In this sense, the available evidence showed how high levels of knowledge about COVID-19 were associated with a positive attitude and correct behaviors [4]. On the contrary, individuals with low levels of knowledge had a lower probability of having the adequate attitudes and preventive behaviors [5].

Health Education (HE) is a useful tool for guaranteeing an adequate level of health-related knowledge, as it promotes behaviors that favor the health of the general population [6]. Also, HE provides the necessary tools for acquiring critical thinking skills, to be able to decide on health matters. Nevertheless, HE goes beyond increasing one's knowledge about personal health-related behaviors, as its intention is to also deploy skills and actions to address determinant social, economic, and environmental factors of health [7]. The first phases of an HE program are the analysis of reality and the detection of needs. Once these are identified, they will allow the design of an intervention adapted to current reality and needs [8].

Thus, after more than two years of pandemic and as evidence was not found in our context, a decision was made to assess the consequences of the pandemic and to delve into the knowledge acquired and that which must be re-enforced. Given that knowledge about this disease has evolved since the start of the pandemic, with the development of specific measures of virus response and containment that are adapted to the WHO guidelines [9]. To provide some context to the situation, at the start of 2022, Catalonia (Spain) was recovering from the seventh wave, and the most dominant variant of SARS-CoV-2 was Omicron, characterized by its fast propagation [10]. According to the Health Ministry of Spain [11], on February 15th, 2022, there was a total of 41,007,734 people with at least one dose of the vaccine (86.6% of the Spanish population), and a total of 38,385,465 with the full vaccination (81.0%).

Given the reasons described above and starting with the following research question—What behaviors were adopted by the general population two years after the COVID-19 pandemic?—the general research objective was to analyze the knowledge, attitudes, and practices associated with COVID-19. More specifically, the secondary objectives of the present study were the following: (a) to identify the knowledge and behaviors related with preventive measures, lifestyle habits, sources of information, vaccination, and the emotions created and coping strategies and (b) to explore the personal experiences related with knowledge, attitudes, and practices associated with COVID-19. This analysis will provide the information necessary to address the lack of knowledge, erroneous ideas, or practices, to therefore modify preventive or health awareness programs [12]. It indispensable for the general population to integrate this information, so that everyone is able to freely, voluntarily, and rationally make health-related decisions [13].

## 2. Materials and Methods

### 2.1. Study Design

The present study is part of the project entitled: *Science-Based Education and Communication to Fight COVID-19 and Future Pandemics* (IlerCOVID). This project is centered on strengthening scientific knowledge about COVID-19 and pandemics in the Segrià region (Catalonia, Spain). It combines four work areas: artificial intelligence, plant biotechnology and neuroCovid, education, and communication. The present study corresponds to the working area of education and includes a HE program based on a brief group education intervention, more specifically on the development of the first phases of the program, in which the knowledge of the population is explored and detailed.

Thus, in line with the objectives described, a study is presented with a convergent mixed methods design [14]. More specifically, the quantitative (QUAN) and qualitative (QUAL) data were collected during a similar amount of time. Afterwards, the two types of data were analyzed separately and in parallel. Lastly, the data were combined, which allowed for a more in-depth exploration of the phenomenon under study.

### 2.2. Context and Participants

The study population in the QUAN part corresponded to individuals 18 years old or older from the different municipalities of the Segrià region. More specifically, according to the data from the Catalonian Statistics Institute, dated January 1, 2021, the total population in the 38 municipalities was 211,609 individuals, of which 81.8% were 18 years old or older (173,010 individuals). The sample size was calculated to estimate a proportion, and given that many parameters were to be assessed, a maximum indetermination (*p*=0.50) position was selected, with a confidence interval of 95%, and assuming a sampling error (*ε*) of 3%, which resulted in a sample of 1,062 individuals.

As for the QUAL part, a purposeful sampling method was utilized [15]. The sampling strategy used maximum variation to achieve sample heterogeneity, based on two aspects: (1) willingness to participate and (2) representation of the general population without excluding any sociodemographic category. Thus, the inclusion criteria were broad in order to obtain diverse and rich information on the phenomenon studied: people older than 18 years, from different age groups, without specifying education or limits according to economic level, and who resided in the Segrià region during the COVID-19 pandemic. As for the exclusion criteria, these were individuals with communication problems, with a language barrier with Catalan or Spanish, or with cognitive problems.

### 2.3. Instruments and Data Collection

For the collection of QUAN data, an ad hoc electronic survey was designed. The variables proposed by the WHO Regional Office for Europe [16] were used. These variables were measured using validated questions or adapted validated questions. The questionnaire as a whole was validated through the six rounds of data collection in Germany. It was translated following the recommendation from the guide itself. Nevertheless, the answers to some of the questions had to be adapted to the country where the study took place, as many of the measures and actions related to COVID-19 were dependent on the national context. Thus, a bibliographic review was performed to adapt the questions to the specific geographical context. Ultimately, 34 questions were included, grouped into 6 sections: (1) sociodemographic data (age, sex, and level of education), (2) COVID-19 preventive measures, (3) lifestyle habits, (4) sources of information, (5) vaccination, and (6) emotions generated and coping strategies. Also, the recommendations on sample sized were followed, as a sample greater than 1000 participants was included. The Pointerpro platform was utilized, and all the city council from each municipality were contacted for the distribution of digital media, during the months of January and February 2022.

In addition, for the collection of the QUAL data, two focus groups were convened in two randomly selected populations in February 2022. Three areas were explored: knowledge, attitudes, and practices (Table 1). The participants were volunteers recruited by the city councils in each municipality through personal contacts and their social networks or dissemination media.

The focus groups met in municipal rooms and were guided by two researchers (JR and CC), an expert senior researcher in QUAL methodology and a novel researcher. These sessions lasted between 90 and 100 minutes. They were audio-recorded for their posterior literal translation. The participants accepted the transcriptions.

### 2.4. Data Analysis

In first place, for the QUAN data, a descriptive analysis of the sample was performed through frequency distribution measurements or measurements of central tendency and dispersion, as a function of the nature of the variables. The statistical program utilized was Statistical Package of the Social Sciences (SPSS) version 27.

In the QUAL analysis of the data, a series of actions were performed to ensure the criteria of credibility, dependability, and transferability [17, 18]: (1) the participants were selected in heterogeneous manner, which provided rich and varied information; (2) information was provided about the participants and the context; (3) at the start, two researchers (JR and CC) revised the units of meaning, the process of abstraction, condensation, and creation of the categories and the topics independently, to increase credibility, and afterwards, in a joint work session with the entire research team, the thematic areas defined in each analysis were agreed upon and discussed; (4) stopping criteria were defined when no new categories emerged in the last focus group [19]; and (5) lastly, the data collected and results found were compared and contrasted in a final report. The analysis was performed with the Atlas-Ti version 8 software.

### 2.5. Ethical Approval

The present study was approved by the Drug Research Ethics Committee from the main hospital in the area (CEIC-2593). The electronic survey participants were informed about the voluntary character and anonymity of the survey, and they were explicitly asked to provide their informed consent before answering the survey. The focus group participants were verbally informed by the researchers and signed an informed consent form. All the data were treated with confidentiality and anonymously.

## 3. Results

The findings from the present study are described in 6 sections (characteristics of the participants, COVID-19 preventive measures, lifestyle habits, sources of information, vaccination, and emotions generated and coping strategies). The QUAN and QUAL data are combined in these sections. See Table 1 in the Supplementary Material for a detailed summary of the QUAL themes, categories/subcategories, and units of meaning.

### 3.1. Characteristics of the Participants

In the QUAN part, 1,559 answers were ultimately obtained (46.8% more than the required sample) from individuals living in the Segrià region, aged between 18 and 90 years old, with a mean age of 49.1 years old (SD = 13.4). Women represented 77.4% of the sample, and 51.1% of the participants had a university degree, followed by secondary (40.7%) and primary (8.2%) education.

As for the QUAL part, a total of 19 participants were obtained (9 from Benavent de Segrià and 10 from Corbins). The age range was 18 to 73 years old, with a mean age of 52.6 years (SD = 19.3). More men participated (57.9%) than women, and most of them had primary education (47.4%), followed by secondary (36.8%) and university (15.8%) education.

### 3.2. COVID-19 Preventive Measures

With respect to the preventive measures, the most recognized by the participants were those related with the use of the face mask (2.3, 2.4, and 2.5), and the least was the option of washing their hands with a hydroalcoholic solution, as shown in Table 2. Only 22.9% identified all the measures that were correct to avoid or reduce COVID-19 infection (^*∗*^ in Table 2).

The participants of the focus groups positively evaluated the use of the face mask, due to its efficacy against other respiratory illnesses (common cold, flu) and affirmed their possible continued use during the transitional period after the pandemic:

“...the mask worked correctly, I think that everyone will use it during winter. The flu and common cold cases have decreased…” BM_4

Other measures listed due to their efficacy against the disease were physical distance and ventilation of the spaces:

“Yes, distancing and ventilation, yes…they were very beneficial…especially ventilation…easy to use.” CM_92

More specifically, with respect to hand-washing, a series of statements were made, which the participants had to identify if they were correct or incorrect. Only 5.1% of the sample correctly defined all the responses (^*∗*^ in Table 3). Below, the responses to each of the statements are detailed (Table 3). It should be underlined that most knew about the importance of hand-washing, despite the virus not being transmitted through contact (95.7%) and that only water was not enough (97.9%). On the contrary, only 25.5% knew that it was necessary to dry their hands so that washing was effective and two thirds of the sample did not know that washing had to be done with water and soap if they were dirty.

On the other hand, according to the other questions related to hand-washing, 82.4% of the participants believed they had enough knowledge for correct hand hygiene, 71.6% affirmed that washing of hands was an act of responsibility, and 66.6% that their hand hygiene habits had improved due to COVID-19, as they became more aware about its importance. As for the frequency, the daily mean of hand-washing was 12.1 (±10.0), oscillating between none to 150 times per day. As for the practices of hand-washing (Table 4), only 21% did so on the necessary occasions, with the most prevalent being after going to the bathroom, before eating, and before preparing any kind of food (higher than 90% of the sample), and the least being before going to the bathroom (39.6%).

According to the QUAL data, the general perception of the participants about hand-washing knowledge was good. However, when exploring this aspect more deeply, some did not know about the efficacy of the products they utilized. Confusion was observed between different hand hygiene terms (washing and disinfection of hands) and the efficacy between the hydroalcoholic solution and soap and water:

“…washing of hands is better with soap and water than with the hydroalcoholic solution…” CM_97

“Anything is useful.” CM_98

It should also be mentioned that only a small number of participants was aware that their hand-washing behavior was not correct:

“We don't wash our hands well!” BM_77

### 3.3. Lifestyle Habits

With respect to the eating habits, the daily mean portions of fruit and vegetables were 3.4 (SD = 1.6), with the weekly mean for legumes being 2.2 (SD = 1.3). As for the consumption of red meat and ultraprocessed foods, about 2 weekly portions (2.1 ± 1.6 and 2.0 ± 2.0, respectively) and sugary drinks were consumed 1.2 ± 2.2 times on average. As for the practice of physical exercise, 56.8% of the sample did so in a planned manner, with a mean of 4 hours per week. And lastly, with respect to toxic habits, 34.1% of the participants had them, with the most prevalent being the consumption of alcoholic drinks (19.6%) and tobacco (18.7%), followed by self-medication (4.7%) and taking narcotics (1.0%).

The QUAL analysis highlighted the presence of changes in the eating habits with these modifications being divergent. On the one hand, the participants expressed a greater consumption of fresh, closer, and healthier foods. This was justified, in part, by the greater availability of time for cooking:

“Fresh fish was consumed... this is good.” CH_10299

“I think that healthier, but we must take into account that we had more time for cooking.” CH_229

And on the other hand, the participants increased food consumption between meals:

“I ate more between meals because I did not leave the house as much.” CH_231

Lastly, on the subject of activity and physical exercise, this habit was promoted as a coping strategy against the negative emotions and feelings that surged during the COVID-19 pandemic:

“Young people started doing physical exercise because we were bored.” CP_213

“In general, people started to go out to walk for leisure, and this habit was maintained.” CP_214

### 3.4. Sources of Information

As for sources of information, as shown in Table 5, the source that was most utilized for obtaining information about the pandemic was the television, more specifically the news or specific documentaries about the subject (43.2%). However, the source of information they considered to be more trustworthy was health professionals, according to more than 70% of the sample studied. Nevertheless, only 35% of the participants utilized the source of information they considered to be more reliable.

In addition, the QUAL findings detected the emergence of the perception that the population was informed, although excessively, causing saturation:

“I'm sorry, but personally, I'm saturated with respect to COVID-19.” CI_120120

“Every day, when I woke up, the media was talking about COVID-19. The information about it was important, but it was excessive…” BI_14

Also, the way the information was transmitted led to difficulties in its interpretation, as it was considered as always changing, incoherent, and lacking in personalization according to age groups and overly sensationalist. On many occasions, this resulted in emotions such as fear:

“They changed the information they provided very often!” CI_114

“…I couldn't leave the house from midnight to six in the morning. So, does the virus come out at night?” BI_12

“The information given was the same, both for young people and for older people. …And the information aimed at youth had to have a motivating purpose.” BI_8

“On television, they showed images that were too sensationalists...” CI_186

“The images shown created fear.” CI_189

### 3.5. Vaccination

As for their state of vaccination (Table 6), 93.8% of the participants was vaccinated, and of these, 66.4% with the third dose. The reasons for becoming vaccinated, from highest to lowest percentage, were as follows: because they believed in science and the vaccines as a prevention method (75.3%), to attain herd immunity (47.2%), because they wanted the pandemic to end (46.2%), to be able to travel and for leisure activities (12.1%), due to social pressure (7.2%), and others such as because they were health professionals or being in contact with at-risk individuals (3.6%). Also, 12.1% believed that the vaccine had some negative effect on their health, with alterations in the menstrual cycle being identified as the most prevalent (50.3%). Of those who were not vaccinated (6.2%), the most common reasons were as follows: they did not trust vaccines (42.7%), because of the risk of adverse reactions due to which the vaccines were greater than the risk of becoming infected (42.7%), and because they had created them too fast (36.5%).

The QUAL data corroborated the ambivalent feelings about vaccination. On the one hand, herd immunity and protection were recognized, and the benefits that the vaccination provided in social life:

“We who are vaccinated increasingly weaken the virus.” BV_21

On the other hand, the vaccines have also created a lack of trust in two levels: one at the economic level and two in relation to the possible adverse effects:

“Of course, it's all about business!” BV_19

“Little has been said about the secondary effects of the vaccine.” CV_128

### 3.6. Emotions Generated and Coping Strategies

With respect to the feelings and emotions generated during the pandemic (Table 7), uncertainty was identified by 47.8% of the participants, followed by fear of the situation (41.8%), sadness (38%), and distress (30.1%), among others. Some of these feelings and/or emotions were identified by 69.5% of the sample, and most identified aspects helped them to manage them (99.1%), such as to talk to their family (38.9%) or their circle of friends (33.1%). However, 52.9% of them still had some of the feelings or emotions described above. As for how they adapted to the new situation provoked by the COVID-19 pandemic, they maintained an optimist or positive attitude (62.3%), social contact (respecting the safety measures) (58.9%), and healthy habits (food, physical activity, and rest) (49.8%), and they also dosed the information on COVID-19 received (49.6%), among others. Lastly, as for the question of if they believed the pandemic, apart from the catastrophe, had contributed something positive, almost 60% said that it had help to put a spotlight on the work of health professionals.

According to the qualitative data, the experiences and the management of the disease could be classified into three categories: the emotions experienced by the participants, their consequences on them, and the techniques utilized to deal with the different situations. With respect to the emotions experienced, the focus group participants classified these emotions as negative as they caused fear, distress, feeling of being unprotected, or uncertainty, although at the same time, they were protective elements:

“At the beginning, everyone was afraid, and the measures were extreme.” CP_185

“There was a lot distress, especially for going outside.” BP_51

“The feeling we had was as if we had been left unprotected!” BP_53

“We didn't know when it would end. Now we are returning to the normal situation, but it is not known if it will last...” CP_191

In a parallel manner, they were able to determine the creation of cooperation as an element of social construction or value:

“The young people, such as us, took the shopping to the elderly, with the help from City Council.” CP_160

“We helped each other.” BP_65

Also, the participants observed experiences during the pandemic in the rural areas that were of higher quality as compared to the cities, due to the type of housing, low-density settlements, life closer to nature (vegetable gardens, fields), and a type of life that was more personal and human:

“I believe that we were privileged during the pandemic, since we lived in a rural area.” BP_36

“The confinement during the month of March was not experienced in the same way as in Lleida or Barcelona. It was very different. Here we were confined, but we were not distressed.” CP_155

The consequences of COVID-19 at the psychological level were characterized by changes in mood during the pandemic, the loss of social relations and loved ones, and a different view of the pandemic according to the different age groups, as well as the before and after marked by COVID-19 in the life of the participants:

“Now we are more relaxed than in the beginning.” CP_200

“Many relationships have been lost…” CP_192

“I missed my grandchildren and not being able to see my daughters.” CP_190

“The children, youth, and elders were the age groups that noted the negative consequences of COVID-19 more strongly than the rest, as they were confined at home” CP_208

“COVID-19 has changed everyone's lives.” BP_52

“It will be hard for us to return to being as before.” CP_203

And lastly, the participants utilized tools such as in-house activities (cook, home improvement, and so on) and social activities from home (drinking vermouth, video-calls), to deal with the emotional situation in which they found themselves in:

“To disconnect from work at the hospital, I began to improve my house.” BP_70

“…each Sunday, we would drink vermouth from the balcony at home, and City Council would play music from the speakers.” CP_161

## 4. Discussion

The present study has allowed the identification of the knowledge and behaviors related with preventive measures, lifestyle habits, sources of information, vaccination, and the emotions generated and their coping strategies, as well as the exploration of the personal experiences with respect to the knowledge, the attitudes, and the practices against COVID-19. Its mixed design has re-enforced the findings obtained, resulting in more profound and detailed knowledge [20]. This is a fundamental aspect for the design of HE interventions, as it must create learning experiences to help develop general skills that are transferable to health [21]. Good knowledge about health allows us to acquire health-promotion behaviors and to act against the challenges posed by COVID-19 [22] or in other possible health situations.

The profile of the QUAN study participants was women older than 50 years with a university degree, in agreement with other recent studies with online questionnaires during the COVID-19 pandemic [23, 24]. However, in the QUAL part, both sexes were equally represented, with a minimal predominance of men and with participants with a low level of education. This could be due to the rural nature of the places where the two focus groups took place. Given the dates and the time in the day of the focus groups (February and in the afternoon), a greater availability could be implied for the male group, as most were farmers, without any other employment obligations.

As for the knowledge of preventive measures against COVID-19, it was shown that they had a good general knowledge, especially related to the 3Ws (wear a mask, wash your hands, and watch your distance). Nevertheless, when specific questions were asked, it was found that they lacked in-depth and specific knowledge about them. One of the most recognized measures was related with the use of the face mask. Also, in our area, the general population had not used it until the arrival of the COVID-19 pandemic. In this sense, the awareness about the use of face masks was more present than other preventive measures [25], perhaps because they produce a false sense of protection, although the mandatory character of their use could have had an influence.

As for hand-washing, only one out of four participants knew that it was necessary to dry one's hands for it to be effective. However, the need for information about the relative efficacy of different drying methods and if drying one's hands completely could have spread SARS-CoV-2 had already been identified [26]. For this reason, the messages from public health authorities should have given the same importance to drying as washing, given the potential for cross-infection, as shown by a recent review on the subject [27]. As for the frequency and the times of hand-washing, the daily mean of hand hygiene events was 12.1 (±10.0), and only 21.1% did so when needed, with these results very similar to other authors [28]. Another aspect that should be highlighted was the lack of knowledge about the efficacy of the hydroalcoholic solution in the prevention of infection and propagation of COVID-19, as compared to water and soap. However, since the outbreak of the disease, its use has increased considerably, and also, the population is more aware about the importance of hand-washing [29].

In general, the lifestyles of the participants were healthy, given that they met the physical activity and diet recommendations [30]. On the other hand, the multiple benefits for mental and physical health of physical activity during the pandemic must be indicated, considering elements such as age, clinical conditions, and level of physical shape [31]. It has been demonstrated that the pandemic contributed towards the maintenance of healthy and active lives, as well as the consumption of fresh foods from nearby areas. Nevertheless, the intake of food during the pandemic was highly influenced by economic factors. Vulnerable individuals experienced food insecurity, which lead to the buying of cheap and less healthy foods, such as packaged and ultraprocessed foods [32]. In agreement with other authors [33], this study has shown that some individuals negatively modified their consumption habits in stressful situations, for example, snacking between meals.

The source from which they obtained more information was the television, specifically news programs or specific documentaries about the subject. However, the source of information they considered to be more reliable was health professionals. This a very positive aspect given that it has been shown that those who trust health professionals with COVID-19 information tend to better adhere to preventive measures [34]. Another aspect that should be highlighted is their perception about an excess of information, which resulted in saturation and the possibility of receiving erroneous or hard-to-interpret information. In this sense, the Director-General of the WHO qualified the situation of disinformation about COVID-19 as an “infodemia” (that is, epidemic of disinformation or pandemic of disinformation), full of conspiracy theories, propaganda, and nonverified scientific statements with respect to the diagnosis, treatment, and prevention of the disease [35]. Therefore, the levels of digital health literacy of the population are also key for the preparation against future infodemias [36], as a low health literacy rate has been shown to result in practices and attitudes that compromise the health of individuals and also the rest of the population due to the proliferation of false information (misinfodemic) [37]. Therefore, it is necessary to consider that eHealth literacy could play an important role in promoting better prevention and control of infections [36]. Nevertheless, despite the messenger (author/source of information) being a fast and reliable indicator of information, the general population must be prepared to evaluate the message (content of information), to improve their levels of health literacy, in light of future infodemias [38].

The vaccination rate of our study sample was high. Nevertheless, there are global variations on the acceptance of the vaccine between populations, although the reasons for vaccination and acceptance of the vaccine are similar. According to a recent systematic review [39], the low acceptance of the vaccine has been associated with low levels of education and awareness and inefficient governmental efforts and initiatives. For this reason, investing in health literacy improves the acceptance of the vaccine and the making of health-related decisions, to reduce the impact of the COVID-19 pandemic [40]. On the other hand, the most prevalent secondary effect was alterations to the menstrual cycle. Recent studies have shown these effects in the shape of premenstrual symptoms (greater fatigue, abdominal distension, irritability, sadness or depression, headaches, and greater difficulty for falling sleep), as well as menstrual changes (higher flows and stronger menstrual pain and shortening of the menstrual cycle) [41, 42].

Some authors [43, 44] corroborate the psychological impact from COVID-19 and the possible negative results for mental health, such as distress, anxiety, and fear, among others. However, the present study did not find more severe states such as depression, suicidal thoughts, or posttraumatic stress disorder (PTSD) [44]. On the one hand, the reasons could be explained with the findings in our focal groups, where it was confirmed that the rural environment seemed to be a protecting factor contrary to the urban areas, which are more complex due to their higher density, and the decreased possibility of maintaining isolation and physical distancing [45]. And, on the other hand, due to the equilibrium between the people who had feelings and/or emotions that could lead to mental health problems (69.5%), and those who used personal tools to cope with them (99.1%). It must be noted that worry or situational anxiety has positive effects, as they lead to preventive behaviors; although if these are persistent, they can result in mental health problems [46]. Also, our results are in agreement with other researchers [47]; when they indicated that people adapted to the guidelines according to the period in the pandemic, they sought ways to compensate for social distancing with other activities (in or outside the home) and other ways to communicate, such as online, or doubling measures of safety (ensuring protection by being outdoors and maintaining distance). In addition, this study shows the cooperation and solidarity during the COVID pandemic at interpersonal levels, by helping the most vulnerable (help nursing homes, older adults, and so on) or vaccinating to attain herd immunity. Lastly, it must be mentioned that the situation of the unprecedented pandemic, the uncertainty, and the risk to become infected are peritraumatic factors that are associated with psychopathology [43]. It is for this reason that 52.9% of the participants still had harbored some of the feelings or emotions mentioned above, without being able to manage them or without being able to find a useful tool to deal with them. Therefore, it is necessary to activate measures organized and centered on the person and based on strategies of active coping, acceptance, and positive thinking, to avoid avoidance strategies to obtain engaged coping that can led to greater well-being [48].

Lastly, the comprehensive study (QUAN and QUAL) of the participants in a particular context allowed us to identify key elements of detection of needs. In this stage examined (two years after the start of the COVID-19 pandemic), key aspects can be inferred about the promotion, education, and health literacy [49]: more resources must be provided to improve community health, it is necessary to provide continuous training in health aspects to the population, and specific strategies must be established so that the population understand the information and act in a manner that is healthy. Also, HE implies the ability to evaluate the information and making decisions to promote protective behaviors [50], but this must be adapted to the context. For this, starting with the results found, it can be specified that (1) the pandemic has sensitized the population, and it is now aware of the importance of individual actions for the common good, for this is a key moment to act; (2) re-enforce the training on basic preventive measures against infectious diseases to achieve behavioral changes that are more integrated and permanent in the population, into day-to-day actions such as hand-washing or the use of protective elements; (3) the information must be integrated as a communicative process and adapted to current channels and the use of technologies; (4) the postpandemic stage has left after-effects in the population at the physical and psychological levels, which must be continuously monitored and addressed, and (5) the lessons learned during the pandemic must help to create a “normality” that is impregnated of the social values that have emerged, such as equity, solidarity, and collectivity. In this sense, the results underline some determining factors of health literacy, but more studies are necessary to understand the impact and the evaluation of health literacy [51]. Nevertheless, the results can serve as the basis for the design of HE interventions, which can act as a modifying factor in health, and have a direct and fast impact on the population [22].

### 4.1. Limitations

Social bias limitations could exist in the data, as the participants could have provided socially desirable responses in relation to the preventive measures. Also, it must be considered that the study was conducted during the postpandemic period (2022), when the experience of the pandemic was still strong, but mediated by the current health situation (vaccinated population and so on). In addition, there could be a bias in the collection of QUAN data online due to Internet access availability. Lastly, the participation in the focus groups was voluntary, and there could therefore be a selection bias, although a broad invitation was performed through the city councils. Nevertheless, the multimethod combination utilized in the present study considerably minimizes these effects.

## 5. Conclusions

This study has allowed the identification of the knowledge and behaviors, as well as the exploration of the personal experiences related with the knowledge, the attitudes, and the practices two years after the start of the pandemic. The results showed that prevention measures must be re-enforced, especially hand-washing, although its importance has already been recognized. However, the use of the face masks is well integrated, and their continued use is expected against respiratory pathologies. Lifestyles remained healthy, through the use of physical exercise as a source of well-being. The population trusted the information from health professionals, but it is excess due to the media, resulted in saturation and negative thoughts. Herd immunity and protection was recognized in vaccination, and although a psychological impact was found in the participants, it was cushioned with the activation of positive coping measures. Nevertheless, more than half of them still maintained it after two years. They must therefore be addressed for the maintenance of mental health.

### 5.1. Implications for Nursing Management

The role of nursing in the management of the COVID-19 pandemic was fundamental. However, studies were not found that performed an in-depth analysis of the knowledge, attitudes, and practices of the population during this time. In this sense, this study allowed the detection of the current needs of the population, with an in-depth analysis of the object of study through the use of a mixed methodology approach. It provides the information needed by nursing personnel to be able to design an intervention adapted to the real needs of the population and to promote HE programs to address COVID-19 or other health situations. Therefore, the present study provides meaningful practical information that could be the starting point for other researchers, nurses and nurse managers and could be used to promote behaviors that favor the health of the general population.

## Figures and Tables

**Table 1:** Areas explored in the focus groups.

Areas	Question asked
Knowledge	What are the preventive measures to deal with COVID-19? And of these, which do you think are the most effective?

Attitudes	What is your opinion about information and communication media? Which of them do you trust more? What is your opinion about the COVID-19 vaccine? What are the reasons of the population for vaccination or not vaccination? How do you assess the impact of the pandemic at the emotional and/or with respect to mental health? What feelings or emotions has COVID-19 generated? Do you think the feelings or emotions at the start of the pandemic are the same as today? Have you overcome these feelings? Or are they still there? To what degree? Do you think the pandemic has provided some positive elements?

Practices	Due to the pandemic, how has your lifestyle changed?

**Table 2:** What measures do you consider important for avoiding or reducing COVID-19 infection?

Measures		Number (*n*)	Frequency (%)
(1) Washing of hands with soap and water^*∗*^	Yes	1240	79.5
	No	319	20.5

(2) Washing of hands with hydroalcoholic solution^*∗*^	Yes	750	48.1
	No	809	51.9

(3) Use of face mask^*∗*^	Yes	1354	86.9
	No	205	13.1

(4) If the mask is not needed, put it under one's chin, hanging from an ear, or the neck, wrist, on top of the head, and so on	No	1527	97.9
	Yes	32	2.1

(5) Removing one's mask to sneeze, to not get it dirty	No	1511	96.9
	Yes	48	3.1

(6) Avoid touching the eyes, nose, and mouth with hands^*∗*^	Yes	916	58.8
	No	643	41.2

(7) Avoid greeting other people with two kisses on the cheeks, hugs, or touching hands^*∗*^	Yes	1007	64.6
	No	552	35.4

(8) Maintain a certain distance (minimum of 1 to 1.5 m) with other people if the mask is not being used^*∗*^	Yes	1219	78.2
	No	340	21.8

(9) Open windows in reduced or closed spaces^*∗*^	Yes	1253	80.4
	No	306	19.6

(10) Reduce social relations when COVID-19 symptoms are detected^*∗*^	Yes	1198	76.8
	No	361	23.2

(11) Vaccinate^*∗*^	Yes	1232	79.0
	No	327	21.0

^*∗*^Correct measure for avoiding or reducing COVID-19 infection.

**Table 3:** Which of the following statements do you think are true with respect to hand-washing?

Statements		Number (*n*)	Frequency (%)
The hydroalcoholic solutions can always substitute hand-washing	Incorrect^*∗*^	1255	80.5
	Correct	304	19.5

How much time is spent when washing one's hands is not important	Incorrect^*∗*^	1317	84.5
	Correct	242	15.5

Hot water increases the effectiveness of hand-washing	Incorrect^*∗*^	1286	82.5
	Correct	273	17.5

It is necessary to dry one's hands after washing for it to be effective	Correct^*∗*^	398	25.5
	Incorrect	1161	74.5

Now, washing one's hands is less important, as it is known that COVID-19 is not transmitted through contact	Incorrect^*∗*^	1492	95.7
	Correct	67	4.3

Washing one's hands with only water is also correct	Incorrect^*∗*^	1527	97.9
	Correct	32	2.1

Hands must be washed with soap and water when they are dirty	Correct^*∗*^	558	35.8
	Incorrect	1001	64.2

^*∗*^Correct answer.

**Table 4:** When do you wash your hands?

Hand-washing occasions^*∗*^		Number (*n*)	Frequency (%)
Before going to the bathroom	Yes	617	39.6
	No	942	60.4

After going to the bathroom	Yes	1488	95.4
	No	71	4.6

Before eating	Yes	1485	95.3
	No	74	4.7

After eating	Yes	941	60.4
	No	618	39.6

When getting home or when going into buildings	Yes	1261	80.9
	No	298	19.1

Before preparing any food	Yes	1437	92.2
	No	122	7.8

After blowing one's nose, cough or sneeze	Yes	1063	68.2
	No	496	31.8

After touching rubbish or money	Yes	1302	83.5
	No	257	16.5

After touching outdoor surfaces	Yes	944	60.6
	No	615	39.4

Before visiting someone who is ill	Yes	1070	68.6
	No	489	31.4

After visiting someone who is ill	Yes	1140	73.1
	No	419	26.9

After touching animals	Yes	1024	65.7
	No	535	34.3

^*∗*^All hand-washing occasions are necessary.

**Table 5:** During the pandemic, where did you get the most information about COVID-19 from, and which do you think is the most reliable source?

Source of information	Where did you obtain most of the information?		Most reliable source	
	Number (*n*)	Frequency (%)	Number (*n*)	Frequency (%)
Social networks (i.e. Instagram, Facebook, and Twitter)	158	10.1	16	1.0
TV: news programs or specific documentaries	674	43.2	223	14.3
TV: dissemination programs	109	7.0	34	2.2
Newspapers or magazines	42	2.7	24	1.5
Health professionals	313	20.1	1119	71.8
Internet	153	9.8	45	2.9
WhatsApp group	16	1.0	5	0.3
Family and friends	47	3.0	17	1.1
Others	47	3.0	76	4.9

**Table 6:** State of vaccination.

		Number (*n*)	Frequency (%)
Participants vaccinated		1463	93.8

Vaccine dose	1 dose	65	4.5
	2 doses	426	29.1
	3 doses	972	66.4

Reason for vaccination^*∗*^	To believe in science and the vaccines as a prevention method	1101	75.3
	To want the pandemic to end	676	46.2
	To attain herd immunity	691	47.2
	Due to social pressure	106	7.2
	To be able to travel and for leisure activities	177	12.1
	Others	53	3.6

Negative health effect	Yes	177	12.1
	No	1017	69.5
	Does not know/does not answer	269	18.4

Why did you have a negative effect?^*∗*^ (*n* = 177)	Because I am more tired	55	31.1
	Because I have a headache or migraines	37	20.9
	Because I have had dermatological alterations	23	13.0
	Because my menstrual cycle has been altered	89	50.3
	Others	53	29.9

Nonvaccinated participants		96	6.2

Reason for nonvaccination^*∗*^	Due to fear	7	7.3
	Lack of trust on the vaccine	41	42.7
	Due to its fast creation	35	36.5
	For fear of needles or shots	2	2.1
	Because of the risk of adverse reactions due to the vaccines was greater than the risk of becoming infected	41	42.7
	Because I think that if I follow a healthy lifestyle, it is enough	22	22.9
	Others	32	33.3

^*∗*^Question with a multiresponse option.

**Table 7:** Emotions generated and coping strategies.

		Number (*n*)	Frequency (%)
Have you felt any of these feelings or emotions during the pandemic: fear, distress, insomnia, stress, anxiety, uncertainty…?	Yes	1083	69.5
	No	476	30.5

What feelings or emotions were generated by COVID-19?^*∗*^ (*n* = 1083)	Fear of the situation	652	41.8
	Fear of going outside	226	14.5
	Distress	470	30.1
	Sadness	592	38.0
	Anxiety	439	28.2
	Insomnia	264	16.9
	Stress	423	27.1
	Uncertainty	745	47.8
	Anger	358	23.0
	Others	28	1.8

Did you use personal tools to cope with them? (*n* = 1083)	Yes	1073	99.1
	No	486	0.9

More specifically, what helped you manage these feelings or emotions?^*∗*^ (*n* = 1083)	Talking to their circle of friends	516	33.1
	Talking to their family	607	38.9
	Talking to health professionals	256	16.4
	Writing	61	3.9
	Practicing some sports	306	19.6
	Others	153	9.8

Presently, do you still have some of these feelings or emotions? (*n* = 1083)	Yes	573	52.9
	No	444	41.0
	Does not know/does not answer	66	6.1

How did you adapt to the new pandemic situation provoked by COVID-19?^*∗*^	Dosing the information on COVID-19 received	774	49.6
	Maintaining the social contact (respecting the safety measures)	919	58.9
	Maintaining healthy habits (food, physical activity, and rest)	776	49.8
	Keeping the mind occupied to not think about the pandemic	445	28.5
	Maintaining an optimist or positive attitude	972	62.3
	Seeking help if needed	180	11.5
	Others	44	2.8

In general, do you believe that the pandemic, apart from the catastrophe, has contributed with some positive lessons?^*∗*^	A greater group feeling	417	26.7
	Better help in the neighborhood	367	23.5
	To help to put a spotlight on the work of health professionals	921	59.1
	To learn the management of health resources	318	20.4
	To be less dependent on primary health care centers	272	17.4
	None of the above (assess it negatively)	237	15.2
	Others	169	10.8

^*∗*^Question with a multiresponse option.

## Data Availability

The participants of this study did not give written consent for their data to be shared publicly, so due to the sensitive nature of the research, supporting data are not available.

## Supplementary Materials

Supplementary Materials Supplementary material has been provided. Table 1 is a detailed summary of the QUAL themes, categories/subcategories, and units of meaning of the findings from the present study.
