# Supplementary material for: Knowledge, Attitudes, and Practices Two Years after the Start of the COVID-19 Pandemic: A Mixed Methods Study
Source: J Nurs Manag. 2024 Feb 16;2024:6636649. doi: 10.1155/2024/6636649 (PMC11919026; doi:10.1155/2024/6636649)
Supplement: Supplementary Materials — Supplementary material has been provided. Table 1 is a detailed summary of the QUAL themes, categories/subcategories, and units of meaning of the findings from the present study. [file 6636649.f1.docx]

Supplementary material, table 1

| **Theme** | **Definition** | **Category** | **Sub-category** | **Unit of meaning** |
| --- | --- | --- | --- | --- |
| COVID-19 preventive measures | To assess the perception of the efficiency of the use of prevention measures against COVID-19 | Usefulness of the mask | Discovery of the efficacy of the mask against other respiratory diseases  (common cold, flu) | “When COVID-19 is over, and if I feel like I have a cold someday, I will use it. It is a measure for not spreading diseases...” CM_99  “In these past years with COVID-19, we haven’t had a cold or the flu” CM_101 |
|  |  | Hand hygiene | Confusion between products | “…washing with soap and water is better than with the hydroalcoholic solution...” CM_97  "Anything works." CM_98 |
|  |  |  | Perception on performing the procedure | “¡We don’t wash our hands well!” BM_77  “I, personally, do not wash them well” BM_78 |
|  |  | Other measures | Distance and ventilation | "Distance." BM_2  "I think ventilation is very important." CM_95 |
| Lifestyle habits | Assess the perception of the changes in lifestyle habits | Consumption of healthy and fresh foods | | "Fresh fish has been consumed." CH_10299  "I think that healthier, but we must take into account that we had more time for cooking" CH_229 |
|  |  | Eating between meals | | "I ate more between meals, because I didn’t leave the house often." CH_231 |
|  |  | Activity and physical exercise | | "Young people started doing physical exercise because we were bored." CP_213  “In general, people started to go out to walk for leisure, and this habit was maintained.” CP_214 |
| Information about COVID-19 | Assess the perception of the information received and the consequences it causes to the receiver | Interpretation of the information | Changing | "They changed the information they provided very often!" CI_114  "Very scattered information, since they said different things on different days." CI_116 |
|  |  |  | Incoherent | “I was released, and they told me I could go to work, but for him, they maintained the confinement, and even extended it! I didn’t understand it...” CI_103  “We could meet for lunch if we went to a restaurant. But, if we organized a meal in a municipal space, we couldn’t do it. And I think it’s the same thing sitting at a restaurant than in municipal space…” CI_106  “…I couldn’t leave the house from midnight to six in the morning. So, does the virus come out at night?” BI_12 |
|  |  |  | Not personalized | “The information given was the same, both for young people and for older people. …And the information aimed at youth had to have a motivating purpose.” BI_8 |
|  |  |  | Excess of information | “Every day, when I woke up, the media was talking about COVID-19. The information about it was important, but it was excessive” BI_14  "I’m sorry, but personally, I’m saturated with respect to COVID-19." CI_120120 |
|  |  | Information and sensationalism |  | “...I think they could approach the message in another way. The images they showed us were too much...” BI_11  "On television, they showed images that were too sensationalists..." CI_186 |
|  |  | Information as the source of fear |  | "The images they showed created fear…" CI_189 |
| Vaccine against COVID-19 | Assess the perception of the vaccination process | Protection and herd immunity |  | “You only need to see the statistics on infections and deaths from COVID-19. These have gone down with the vaccines...” CV_126  "We who are vaccinated increasingly weaken the virus." BV_21 |
|  |  | Generation of distrust | Economic | "It is a business, where the creators of the vaccine receive subsidies, or..." BV_17  “Of course, it’s all about business!” BV_19 |
|  |  |  | Possible adverse effects | "Little has been said about the secondary effects of the vaccine." CV_128 |
| Psychological impact of COVID-19 | Experience and management of COVID-19 at a psychological level | Emotions experienced | Fear | "The level of fear we had surprised me." BP_48  “In these last two years, most people have not travelled due to fear…” BP_49  "At first, everyone was very afraid, and the measures were taken to the extreme." CP_185 |
|  |  |  | Distress | “At first everyone felt distressed...” BP_50  “There was a lot distress, especially for going outside.” BP_51 |
|  |  |  | Feeling of being unprotected | “The feeling we had was as if we had been left unprotected!” BP_53  “What was experienced very negatively was the way these people died. And also, how their families had to cope.” CP_218 |
|  |  |  | Uncertainty | “We didn't know when it would end. Now we are returning to the normal situation, but it is not known if it will last...” CP_191 |
|  |  |  | Cooperation | “We made masks with clothes we had at home. And the City Council coordinated it.” BP_38  “People took cell phones that they did not use in the residences. Thus, residents could make video calls with their families.” CP_162  "The young people, such as us, took the shopping to the elderly, with the help from City Council." CP_160  "We helped each other." BP_65 |
|  |  | Consequences | Missing family | “I missed my grandchildren and not being able to see my daughters.” CP_190 |
|  |  |  | Adaptation of emotions to the pandemic | "Now we are more relaxed than at the beginning." CP_200  “At first, we were all more afraid. Instead, we have now relaxed and become more relaxed.” CP_202 |
|  |  |  | Losses | "Many relationships have been lost." CP_192  "Meetings with friends are over and also meetings in the nursing homes." CP_194 |
|  |  |  | Difficulty returning to the old normality | “Now it will be difficult for us to resume some activities, such as meeting friends or going on a trip.” BP_69  "It will be hard for us to return to being as before." CP_203 |
|  |  |  | Differences by age groups: children, adolescents and older adults | "The children did not receive psychological or emotional support." BP_54  “...older people are afraid to go out again.” BP_57  “Adults have continued to work. That is why children and adolescents had the worst time as a group.” CP_205 |
|  |  |  | Life changing | "COVID-19 has changed everyone’s lives." BP_52 |
|  |  | Coping | Benefits of rural life | "I believe that we were privileged during the pandemic, since we lived in a rural area." BP_36  “The confinement during the month of March was not experienced in the same way as in XXX or XXX. It was very different. Here we were confined, but we were not distressed.” CP_155 |
|  |  |  | At-home activities (cooking, home improvement, etc.) | “We have cooked more because we had to pass the time in some way. And I like to cook.” CP_220  "To disconnect from work at the hospital, I began to improve my house." BP_70 |
|  |  |  | Social activities from home (drinking vermouth on the balconies, video calls, etc.) | "...each Sunday, we would drink vermouth from the balcony at home, and City Hall would play music from the speakers..." CP_161  “...you make a video call with another person and that’s how you entertain yourself...” CP_216 |
